# Influence of Dispersing Method on the Quality of Nano-Admixtures Homogenization in Cement Matrix

**DOI:** 10.3390/ma13214865

**Published:** 2020-10-30

**Authors:** Elżbieta Horszczaruk, Paweł Łukowski, Cyprian Seul

**Affiliations:** 1 Department of Reinforced Concrete Structures and Concrete Technology, Faculty of Construction and Environmental Engineering, West Pomeranian University of Technology in Szczecin, 71-310 Szczecin, Poland; 2 Department of Building Materials Engineering, Faculty of Civil Engineering, Warsaw University of Technology, 00-637 Warsaw, Poland; p.lukowski@il.pw.edu.pl; 3 Department of Geotechnics, Faculty of Construction and Environmental Engineering, West Pomeranian University of Technology in Szczecin, 71-310 Szczecin, Poland; cyprian.seul@zut.edu.pl

**Keywords:** cement composite, dispersing methods, nano-modifiers

## Abstract

In recent years, a nano-modification of the cement composites allowed to develop a number of new materials. The use of even small amount of nano-admixture makes possible not only to improve the physico-mechanical properties of the cement materials, but also to obtain the composite with high usability, optimised for the given application. The basic problem of nano-modification of the cement composites remains the effectiveness of dispersing the nanomaterials inside the cement matrix. This paper deals with the effect of the type and size of the nanoparticles on the tendency to their agglomeration in the cement matrix. The main techniques and methods of dispersing the nanomaterials are presented. It has been demonstrated, on the basis of the results of testing of three nanomodifiers of 0D type (nano-SiO_2_, nano-Fe_3_O_4_ and nano-Pb_3_O_4_), how the structure and properties of the nanomaterial affect the behaviour of the particles when dissolving in the mixing water and applying a superplasticiser. The nanoparticles had similar size of about 100 nm but different physico-chemical properties. The methods of dispersing covered the use of high-speed mechanical stirring and ultrasonication. The influence of the method of nano-modifier dispersing on the mechanical performance of the cement composite has been presented on the basis of the results of testing the cement mortars modified with 3% admixture of nano-SiO_2_.

## 1. Introduction

The use of various nano-admixtures became, in the recent decade, one of the most popular methods of creating the innovative cement composites. Modification of the cement composites with nanomaterials allows not only to improve the mechanical performance of the composites, but also to obtain new, unparalleled features, like electric conductivity, self-cleaning or even self-repairing ability [1]. The basic problem in the nano-modification is effectiveness of nanomaterials dispersion in the cement matrix. Various modifiers, including nano-admixtures, are added to the dry components of the composite in the form of the water dispersion. The nanoparticles mixed with water demonstrate a tendency to agglomeration due to van der Waals forces. Therefore, one of the main obstacles in preparing the cement nano-composite is difficulty in obtaining the mixture with homogeneously dispersed inclusion [2]. The commercial nanoparticles, in spite that they are produced with the size below 100 nm, after application to the cement composite create usually large agglomerates with size from 1 to 10 mm [3,4,5]. The agglomeration of the nanoparticles in the cement matrix can lead to creation of the weak zones with increased porosity, and, consequently, to the poorer mechanical performance of the composite [4,5]. Therefore, the “ideal” dispersing of the nanoparticles in the cement matrix became the highest aim for the researchers involved. This covers the study of the factors, which can even to a small extent counteracting the agglomeration of the nanoparticles.

In the majority of the research works, the nanoparticles are dispersed in the water environment, often in the presence of the superplasticiser [5]. Less often, the nano-modifier is applied directly to the cement [6]. These environments are significantly different regarding both the methods of nanoparticles application and the final effects. The quality of dispersing is usually evaluated on the basis of performance of the modified composite, e.g., the improvement of its mechanical properties. 

There are also researches reported, in which the quality of nanoparticles dispersing in the matrix was evaluated by various methods—for instance, the scanning electron microscopy (SEM) and transmission electron microscopy (TEM). These investigations concern mainly the carbon nanotubes and nano-fibres [7,8,9].

There are sodium, potassium, calcium, sulphate and hydroxylic ions presented in the hardening cement paste together with less concentrated aluminium and silicon ions [10]. The concentrations of these ions are changing during the cement hydration. During the first hours after mixing the concentration of the calcium, sulphate ad hydroxylic ions remains stable. After 6 to 24 h, the concentration of the calcium and sulphate ions begins to diminish, while the concentration of the hydroxylic, silicon and aluminium ions is growing [11]. Moreover, when the superplasticiser is present, it also can increase the content of the sodium and hydroxylic ions, depending on the superplasticiser amount. The knowledge of these processes can be important for the finding the proper way of the nanoparticles dispersing in the cement matrix. The key phenomenon here, besides the action of van der Waals forces, seems to be the effect of Ca^2+^ ions, the concentration of which is responsible for the nanoparticles agglomeration. For example, the behaviour of the nano-silica dispersion in the cement paste is influenced by the Ca^2+^ bridging effect. Thus, the ionic composition of the cement systems seems to significantly affect the distribution of nano-silica particles [12]. Both the concentrations of the ions in the pore solution as well as the tendency of the nano-silica particles to adsorb affect the stability of SiO_2_ nanoparticles and can result in the nano-particles agglomeration [13,14].

The effect of pH value can also be important for the nanoparticles dispersing. In the case of multi-walled carbon nanotubes (MWCNT), functionalised by Ca(OH)_2_ powder, the increase of pH value led to diminishing the zeta-potential of the nanoparticles [15]. This made the interaction between the functional groups of MWCNT and the superplasticiser more difficult and caused the re-agglomeration of the nanoparticles. The measurement of zeta-potential can be useful then; it is most often performed using the method of the laser Doppler effect during electrophoresis (laser Doppler electrophoresis, LDE).

Obtaining an “ideal” dispersion of nanoparticles requires breaking the agglomerates to the smaller parts, even to the original size of the particles using the necessary amount of energy. After breaking the agglomerates, the next step is stabilisation of the “broken” parts and prevention of their reagglomeration using the suitable mechanism, e.g., the steric or electrostatic repulsion. The typical mixing of the components is not sufficient for obtaining the best dispersion of the nanoparticles in the cement composite, even at the high rate of mixing. For this aim the combination of the mechanical methods and chemical modifications is used. These methods are used separately or simultaneously, depending on the type of nano-modifiers and environment. 

The mechanical methods, including mechanical mixing [16,17], ultrasonication [18] and ball grinding [4], are widely used for dispersing the nanoparticles in the water suspensions and dry binders. The most often used mechanical methods are listed and briefly characterised in Table 1. The physical modifications of the nanoparticles surfaces with organic admixtures [19,20] and various types of surfactants (e.g., ethanol) [21,22] are used for making the dispersing of the nanoparticles easier. The chemical treatment of the nanoparticles surfaces—functionalisation—is also employed for this aim [23,24]. In the industrial production of the cement composites, the most often used modifiers are the superplasticisers [25]; they are necessary, for instance, to produce the high-strength concrete. Superplasticisers are also the most common dispersing means for the 0D nanoparticles (i.e., the nanoparticles with all three dimensions below 100 nm), including nano-SiO_2_ [26]. Using the silica nanoparticles in the cement paste without a superplasticiser leads to increase of the mixture viscosity and introduce additional air bubbles to the matrix [27], which is harmful for the porosity structure. Below, the most often used methods of dispersing the nanomaterials with various structures, most often used for modification of the cement composites, are presented.

Nano-silica (nano-SiO_2_) is the best known active (reacting with cement components) nano-modifier of 0D type. The size of SiO_2_ nanoparticles is important for their dispersing and affects the composite properties. The nanoparticles with small initial size (10–20 nm) demonstrate strong tendency to agglomeration and formation of the large aggregates [5]. SiO_2_ nanoparticles with larger diameters (above 40 nm) are better dispersed and affect the mechanical properties of the cement pastes and mortars in more favourable way [28]. The method of nanoparticles synthesis is important factor affecting the final size of the particles and their tendency to agglomeration. The commercial SiO_2_ nano-powders, although designed for the primary particle size of nanometers, usually consist of the large aggregates with the size of micrometers [3]. The most often used methods of nano-silica dispersing are ultrasonic bath and high-speed mechanical mixing [29,30] as well as high-pressure homogenisation [31]. Dispersing the nano-SiO_2_ is conducted in water, often with an admixture of the superplasticiser [5,32].

Graphene oxide GO is a typical example of a 2D nanomaterial used in cement composites. The main problem with the use of graphene in the cement composites is its poor dispersing ability in water [33]. In order to obtain the nano-GO modified cement composite, it is necessary to exfoliate the graphene sheets, and then disperse the flakes, first in the water and next in the cement matrix [34]. The direct exfoliation of the graphene by sonication helps to maintain the basic shape of the GO flakes and prepare them before mixing. The excessive sonication, however, can negatively affect the size and shape of GO flakes, deteriorating their structure [35]. In some studies, the mechanical mixing was employed as the initial stage of GO dispersing. Then, the additional methods, like the use of ultrasounds [36] or mixing after adding the dispersing solution prepared by ultrasonication (ultrasonic bath) [37], were used. Many researchers have used ultrasonication as the main approach to production of the water dispersion of graphene and its derivatives [38]. Recommending the optimum parameters of ultrasonication regarding to time and power is difficult due to the little information available. The use of superplasticisers in combination with ultrasonication appeared to be positive for both the quality of GO dispersing and shortening of the sonication time. The superplasticisers also compensate the common side effect of using the nanomaterials, namely the worsening of the cement composites workability [39]. The well-dispersed graphene suspensions require the resonication of the ready composite mixture for maintaining the long-term homogeneity [40]. The chemical modification of graphene by oxidation allows to its better dispersing in water [41]. This method, however, also has significant disadvantages regarding to the use of this nano-filler in the cement composite. GO loses some of its advantages, including the electric conductivity. The oxidation causes creases in the sheets, increasing the binding potential [41].

The typical example of 1D nano-modifier used for the modification of the cement composites are the carbon nanotubes (CNT). The carbon nanotubes are very good strengthening for the nano-composites thanks to the ultra-high strength—about 60 GPa, Young’s modulus—about 1 TPa, total deformation—12% [42] and shape coefficient. CNT can be single-walled, SWCNT, or multi-walled, MWCNT. SWCNT consist of one sheet of graphene rolled up into long, empty cylinder, while MWCNT are nested graphene tables [43]. SWCNT are more effective due to the better contact between the fibres and the cement matrix because of higher relation of the surface area to the volume [44]. The sufficient dispersing of SWCNT, however, is more difficult to achieve due to their strong tendency to agglomeration. Therefore, the multi-walled nanotubes are more frequently chosen in the case of the cement composites, as manufacturing the composite and dispersing the nanotubes is easier and the production is generally less expensive [45,46].

Various methods are used for CNT dispersing in the cement matrices, including mechanical methods, like ultrasonication or magnetic stirring, as well as chemical methods, e.g., adding the surfactants and functionalisation.

Functionalisation of CNT is an important process allowing to practical use of this nano-modifier in many areas. Chemical modifications of CNT lead to significant changes of their physico-chemical behaviour (e.g., hydrophilic–hydrophobic and acidic–alkaline properties) as well as electrochemical and catalytic features. The carbon nanotubes functionalised with oxygen or nitrogen groups can be apply as intermediate product for creating more developed structures, e.g., with polymers, which are often used for cement composites modification [47]. The superficial nitrogen functional groups change the structure of electrons in the carbon nanotubes; this leads to the localisation of the charge density, important for the reactions running with charge transferring [48]. Because of their specific properties, CNT play an important role in catalysis and sensorics. The most often used admixtures intended for CNT dispersing in the cement matrix are polycarboxylate superplasticisers [49]. Joining the functional groups –COOH to the CNT surface improves the bonding between CNT and the cement matrix with simultaneous weakening the van der Waals forces [19]. The carboxyl groups cause the electrostatic repulsion between CNT, but the main mechanism is here the steric repulsion resulted from the long polyether side chains [50]. 

The motivation for preparing this paper was investigation of the influence of the structure and properties of the selected nano-admixtures on their dispersing in water in the presence of the superplasticiser. Three commercially available nano-admixtures of 0D type with the diameters of about 100 nm, having different physico-chemical properties, were selected for the studies. The first one was nano-SiO_2_, which is an active nano-filler in the cement paste environment, as it affects the cement hydration process [12]. The other two were the metallic nano-modifiers, inactive in the presence of cement: nano-magnetite (nano-Fe_3_O_4_) with the strong magnetic properties and nano-Pb_3_O_4_. Additionally, for analysing the influence of the method of nano-modifier dispersing on the mechanical performance of the cement composite, the tests of the cement mortars modified with 3% admixture of nano-SiO_2_ were conducted, in which the nano-modifier was applied to the composite by three different methods. It has been demonstrated that the method of the simultaneous mechanical stirring and ultrasonication allows to prevent the agglomeration of the tested nano-admixtures in the effective and durable way. The use of the superplasticiser stabilises the nano-admixture’s dispersion in water, making re-mixing unnecessary.

## 2. Materials and Methods

### 2.1. Nanomaterials and Chemical Admixtures

Investigation was conducted with the use of commercially available nanomaterials:

- nano-SiO_2_, nano-silica—dispersion of colloidal silica with approximately 50% solids by weight, the particles were spheres with the size 40–140 nm, density 1.4 g/cm^3^, viscosity 8 cP, pH 9.5 (product of Levasil OF8);
- nano-Fe_3_O_4_, nano-magnetite—the particles have a cubic structure with the size of about 50–100 nm, purity 97% (product of Sigma Aldrich 637106, (Sigma Aldrich, Saint Louis, MO, USA);
- nano-Pb_3_O_4_—the particles were spheres with the size 1–2 µm, purity 99% (product of Sigma Aldrich 241547).

A polycarboxylate superplasticiser (Master Glenium SKY 686, BASF, Myślenice, Poland) was also used.

### 2.2. Cement and Fine Aggregate

Ordinary Portland Cement (OPC) type 42.5R was used as the binder material, conforming to the requirements of BS- EN 197-1 [51]. The chemical compositions and physical properties of the cement used are shown in Table 2. The quartz sand with the maximum grain size 2 mm and the specific density 2.64 g/cm^3^ was used for preparing the tested mortars.

### 2.3. Mixtures Proportioning and Preparation of Mortar Specimens

The SiO_2_ nanoparticles have been selected to the further tests due to their active participation in the process of cement hydration [12]. For determining the mechanical properties of cement, the following mortars were prepared: R—reference mortar and A, B, C—mortars containing 3 wt% of solid nano-silica particles, prepared using three various methods. The composition of the cement mortars is presented in the Table 3.

In method A, the nano-SiO_2_ in the form of powder was added to the cement and mixed for 5 min in the mortar mixer, then the sand and water were added and mixed according to the procedure given in BS-EN 197-1 [51]. In method B, the nano-SiO_2_ in the form of 50% water suspension (commercially available) was initially mixed mechanically with the mixing water (mixing rate 2200 rpm) and simultaneously ultrasonicated for 10 min (frequency 20 kHz). Such obtained water dispersion of nano-SiO_2_ was added to the dry components of the mortar. In method C, the nano-SiO_2_ in the form of 50% water suspension was mixed with the water containing 1.5% of polycarboxylate superplasticiser; the mixture was mechanically mixed (mixing rate 2200 rpm) and ultrasonicated for 10 min (frequency 20 kHz). Such obtained water dispersion of nano-SiO_2_ was added to the dry components of the mortar. Next, the fresh mortar was poured into oiled moulds to form the specimens with a size of 40 mm × 40 mm × 160 mm, according to the requirements of BS-EN 196-1 [52]. The samples were demoulded after 24 h and then cured for 2 and 28 days in the standard water bath at a temperature 20 °C ± 2 °C. The specimens were then dried and mechanical tests were carried out.

### 2.4. Test Methods

#### 2.4.1. Dispersing the Nanomaterials in Water

Determination of the particle size distribution is an effective tool for improving the nanoparticles dispersing in water. Mastersizer 2000 (Malvern Instruments Ltd., Malvern, UK) was used to measure the particles size by laser light scattering method. The device allows to measure the particle size within the range from 0.0001 mm to 2 mm.

Investigation of nanomaterials dispersing in water has been divided into two stages. The first stage consisted in determination of time necessary for permanent breaking the nano-admixture agglomerates. The second stage covered the testing of the impact of superplasticiser on the distribution of the nanoparticles size in water dispersion.

In the first stage, every sample of nano-admixture was dispersed in 700 mL of the water with the properties compliant with the European Standard EN 1008 [53] and mixed mechanically at the rate 2000 rpm for 60 s. Then the obtained dispersion was ultrasonicated at the frequency 20 kHz for 5 min. The time of initial break of the samples has been accepted on the basis of the numerous investigations, presented by various authors [6,34,46,48]. Then, after measurement, the dispersion was again mixed mechanically for 60 s and ultrasonicated for the next 5 min. After this, the next measurement was performed. The distribution of the size of nanoparticles was determined three times for each sample.

In the second stage, the obtained dispersion of nano-admixture was further treated by adding 5 mL of superplasticizer and mechanical mixing for 60 s. Again, the size distribution was determined and after this the dispersion was ultrasonicated at the frequency 20 kHz for 5 min. The last measurement (also three times for each sample) was conducted after this final breaking.

#### 2.4.2. Mechanical Properties of the Mortars

After 2 and 28 days of curing, the compressive and flexural strength of the specimens were tested in accordance with BS-EN 196-1 [52].

## 3. Results and discussion

### 3.1. Dispersing of the Nanomaterials in Water

**Figure 1:**
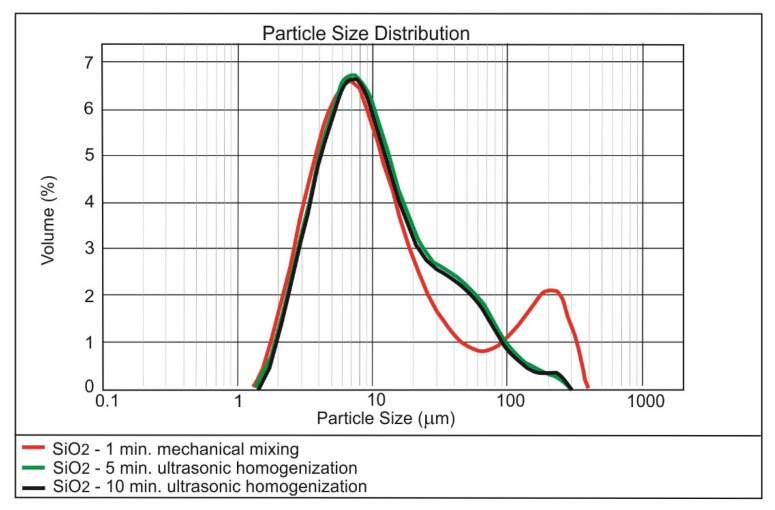
Effect of ultrasonic dispersing on the nanoparticle size distribution of SiO_2_ in the water dispersion.

**Figure 2:**
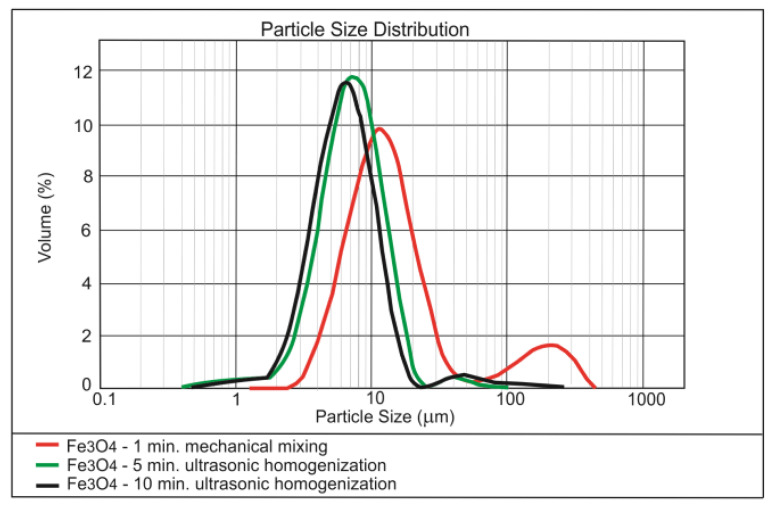
Effect of ultrasonic dispersing on the nanoparticle size distribution of Fe_3_O_4_ in the water dispersion.

**Figure 3:**
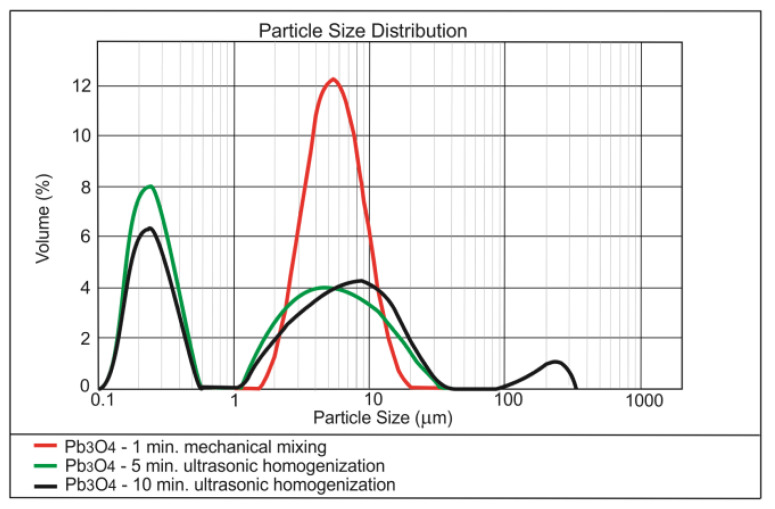
Effect of ultrasonic dispersing on the nanoparticle size distribution of Pb_3_O_4_ in the water dispersion.

The results of testing the effect of dispersing time on the nanoparticles size distribution are presented in Figure 1, Figure 2 and Figure 3.

Analysis of the Figure 1, Figure 2 and Figure 3 shows that 5 min of ultrasonication is sufficient for the long-term homogenisation of the nano-modifiers in the mixing water. The commercial nano-silica has been permanently dispersed, which is demonstrated by the stability of the main granulometric peak regardless of the time of ultrasonication (Figure 1). Poor homogenisation of the nano-Fe_3_O_4_ (Figure 2) was mainly a consequence of the magnetic properties of the nano-admixture; this issue was discussed in detail in [52]. In the case of nano-Pb_3_O_4_, the best dispersing in water was achieved by ultrasonication (Figure 3).

**Figure 4:**
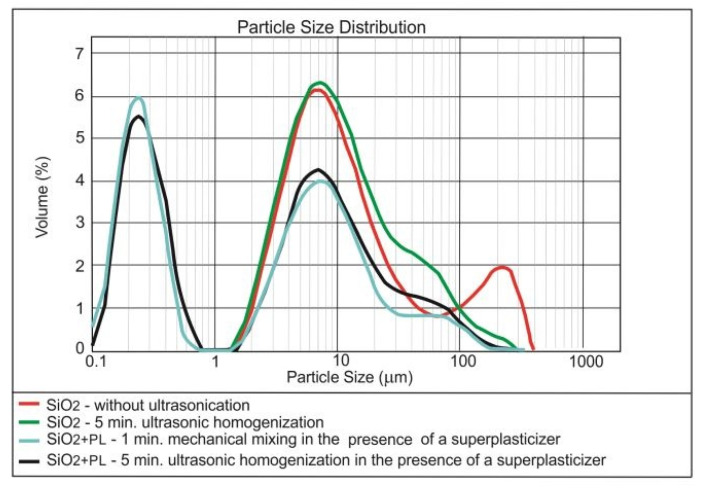
Effect of ultrasonic dispersing on the nanoparticle size distribution of SiO_2_ in the water dispersion in the presence of the superplasticiser.

**Figure 5:**
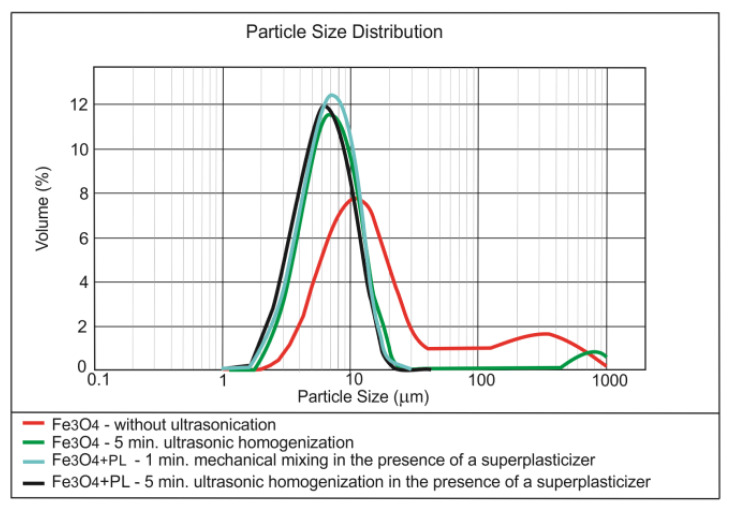
Effect of ultrasonic dispersing on the nanoparticle size distribution of Fe_3_O_4_ in the water dispersion in the presence of the superplasticiser.

**Figure 6:**
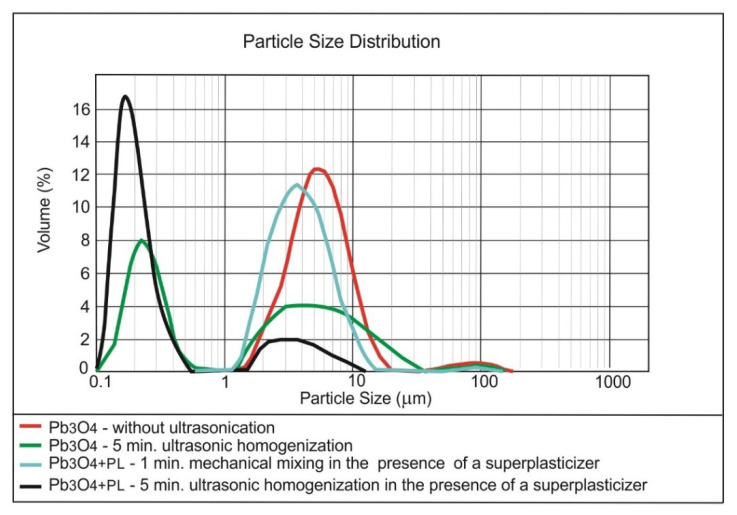
Effect of ultrasonic dispersing on the nanoparticle size distribution of Pb_3_O_4_ in the water dispersion in the presence of the superplasticiser.

Figure 4, Figure 5 and Figure 6 present the results of testing the effect of dispersing time on the nanoparticles size distribution in the presence of the superplasticiser.

The superplasticiser admixture, added to the nano-SiO_2_ initially dispersed in the water by ultrasonication, caused further fragmentation of the nano-silica agglomerates (Figure 4). The re-application of the ultrasounds, however, did not resulted in the better breaking of SiO_2_ nanoparticles. In the case of nano-Fe_3_O_4_, adding the superplasticiser and further ultrasonication did not improve the nanomaterial dispersing (Figure 5); the superplasticiser only improves the cement composite workability. The mechanical mixing with the use of the magnetic stirrer can prove to be more effective for the nanoparticles with the magnetic properties [52]. The superplasticiser clearly caused the further breaking of nano-Pb_3_O_4_ agglomerates (Figure 6). Further ultrasonication, however, did not improve the nanomaterial dispersing.

Breaking the agglomerates of nano-admixtures in the water dispersion by combination of ultrasonication and high-speed mechanical mixing seems to be the most effective way of dispersing the nano-modifiers in the mixing water. However, there is still little information available about the details of time and frequency used at the ultrasonication as well as the rate of mechanical mixing.

### 3.2. Mechanical Properties of Mortars

The results of testing of the flexural and compressive strength of the mortars are presented in the Table 4.

**Figure 7:**
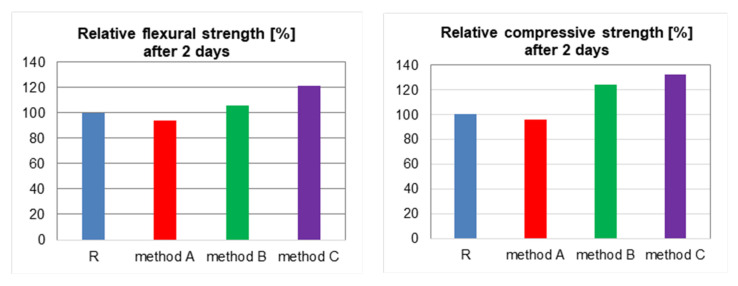

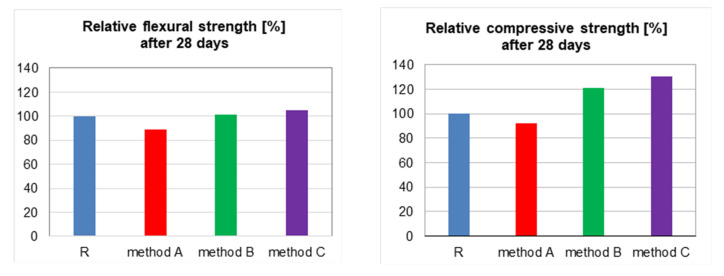
Effect of the nano-SiO_2_ dispersing method on the mechanical properties of the cement mortars (the methods are described in the text above).

The results of tests show that the least effective method of applying the nano-admixtures is the standard method [52] using the conventional mechanical stirring of the dry components of the mortar. In the case of the mortar prepared by method A, even the downfall of the strength was observed. The flexural strength of the specimens prepared by the method A decreased by 6% after 2 days of curing and by 11% after 28 days of curing, as compared to the reference mortar. The compressive strength decreased by 4% and 8%, respectively. According to the literature [6,53], the presence of the places with significant agglomeration of nano-SiO_2_ causes an increase of the cement matrix porosity. This, in turn, leads to the decrease in the strength of the composites. An improvement of the mechanical performance of the mortars was achieved when the nano-silica was deagglomerated by simultaneous mechanical stirring at the high speed and ultrasonication (method B). The most effective method for the tested mortars appears to be method C, where the superplasticiser allowed for more effective dispersion of the agglomerates. This has been confirmed by the tests results presented in the Figure 4. The flexural strength increased by 21% after 2 days of curing and by 5% after 28 days of curing, as compared to the reference mortar, while the compressive strength increased by 30% after both 2 and 28 days of curing (Figure 7). 

## 4. Sum Up and Conclusions

The investigation presented in the paper, conducted with the use of three nano-admixtures with the similar particle size, but different physico-chemical properties, show the significant influence of these properties on the nano-modifiers dispersing in water. The superplasticiser added after initial mechanical stirring and ultrasonication improved the quality of dispersing. The fluidising admixture covers the dispersed nanoparticles, and thus prevents their re-agglomeration, additionally improving the cement composite workability [6,54]. The tests results confirmed that the time of ultrasonication during the initial mixing of the nano-admixture with water can be shortened to 5 min.

The tests carried out on the cement mortars modified with 3% of nano-SiO_2_ confirmed the effectiveness of the dispersing method using the simultaneous mechanical stirring at the high speed and ultrasonication in the presence of the superplasticiser. An increase of the compressive strength of the mortar by 30%, as compared to the reference mortar, was achieved when using this method.

The geometrical parameters and the physico-chemical properties of the nanoparticles play the crucial role in their agglomeration and affect the possibility of achieving the proper nano-admixture dispersion in both water and cement matrix. The 0D nanoparticles are easier to disperse in the cement matrix because they are less complex than 1D or 2D nanomaterials. Dispersing the 1D nanomaterials, like carbon nanotubes, is more difficult and requires more energy due to their geometrical complexity, which leads to internal joining and entanglement. Dispersing the 2D nanoparticles is even more complicated due to their higher surface energy and stronger tendency to agglomeration [55]. There is the preferred method of dispersing for each type of nanoparticles, and each of these methods requires specific conditions for achieving the best possible dispersing.

The desirable dispersion can be obtained by two-stage procedure covering several steps. The first stage of dispersing consists in breaking the agglomerates formed during and after nanoparticles synthesis or in dispersion. Various mechanical methods can be applied for this aim; their effectiveness depends mainly on the degree of agglomeration and bond strength. There is lack of consensus concerning the optimum values of the power, time, amplitude and other parameters of the mechanical methods used in the first stage of dispersing. The second stage covers chemical modification of the nanoparticles surface that leads to the stabilisation of dispersion and prevention of re-agglomeration.

The combination of the mechanical methods (e.g., ultrasonication and/or high-speed mixing) with the chemical modification (functionalisation or coating) allows achievement of the effective dispersion of nanomaterials in the cement matrix. It should be noted, however, that there is still no effective method developed for stopping the agglomeration after adding the nano-admixture dispersion to the concrete mix.

## Figures and Tables

**Table 1:** Comparison of mechanical methods of deagglomeration of nanomaterials.

Method	Form of Nanoparticles	Mechanism	Advantages	Disadvantages
Grinding	Powder	Grinding as fine as possible	The method is useful for the large volumes of nanoparticles	The method is slow and low-efficient
Magnetic and mechanical stirring	In liquid media	The stirrer rotating at the high speed, sufficient to create a vortex	The method is relatively inexpensive	The method is low-effective, re-agglomeration usually occurs when stopping to stir
High-speed homogenisation	In liquid media	High rate of rotation draws the material to the head, where it is mixed intensively The centrifugal force directs the material to the edges of the head, where it is mechanically cut inside the gap between the rotor and the stator	The method is useful for the large volumes of the liquids	The method is rarely used, mainly for the graphene oxide (GO) and other 2D nanomaterials
High-pressure homogenisation	In liquid media	Increase of the velocity of liquids streams under pressure inside the micro-channels causes shear and cavitation	The method is highly effective	The method usually causes the rise of temperature and is expensive
Ultrasonication	In liquid media	Use of the ultrasonic waves energy and cavitation in the water	The method is relatively inexpensive	The method can change the nanoparticles structure due to the rise of temperature. Efficiency of the process is unstable

**Table 2:** Chemical compositions and physical properties of cement [wt.%].

Cement Type	CaO	SiO_2_	Al_2_O_3_	Fe_2_O_3_	MgO	Na_2_O	K_2_O	SO_3_	Loss on Ignition LOI	Specific Density [g/cm^3^]	Surface Area [cm^2^/g]
CEM I 42.5 R	63.29	19.52	4.88	2.91	1.31	0.10	0.90	2.79	3.01	3.05	3956

**Table 3:** Mix proportions of mortars.

Mix Designation	Sand [g]	Cement [g]	Water [g]	Water/Cement	SiO_2_ [g]	SiO_2_/Cement	Superplasticiser/Cement
R	1546	518	257	0.5	0.00	0.0%	0.0%
Method A	1546	518	257	0.5	15.6	3.0%	0.0%
Method B	1546	518	257	0.5	15.6	3.0%	0.0%
Method C	1546	518	257	0.5	15.6	3.0%	1.5%

**Table 4:** Flexural and compressive strength (mean value of three specimens) of mortars after 2 and 28 days of curing.

Sample Designation	R	Method A	Method B	Method C
Flexural strength after 2 days, MPa	5.70	5.36	6.04	6.89
Flexural strength after 28 days, MPa	7.62	6.78	7.70	8.01
Compressive strength after 2 days, MPa	31.53	30.27	39.09	41.62
Compressive strength after 28 days, MPa	52.18	48.01	63.14	67.83
